# ArrayD: A general purpose software for Microarray design

**DOI:** 10.1186/1471-2105-5-142

**Published:** 2004-10-02

**Authors:** Anu Sharma, Gyan Prakash Srivastava, Vineet K Sharma, Srinivasan Ramachandran

**Affiliations:** 1 Institute of Genomics and Integrative Biology (CSIR), Mall Road, Delhi 110 007, India

## Abstract

**Background:**

Microarray is a high-throughput technology to study expression of thousands of genes in parallel. A critical aspect of microarray production is the design aimed at space optimization while maximizing the number of gene probes and their replicates to be spotted.

**Results:**

We have developed a software called 'ArrayD' that offers various alternative design solutions for an array given a set of user requirements. The user feeds the following inputs: type of source plates to be used, number of gene probes to be printed, number of replicates and number of pins to be used for printing. The solutions are stored in a text file. The choice of a design solution to be used will be governed by the spotting chemistry to be used and the accuracy of the robot.

**Conclusions:**

ArrayD is a software for standard cartesian robots. The software aids users in preparing a judicious and elegant design. ArrayD is universally applicable and is available at .

## Background

Microarray is a popularly used high-throughput technology to investigate gene expression of thousands of genes simultaneously at the level of mRNA. Ever since the development of this technology [1-3], transcriptional profiling at the genomic level has been employed to address numerous issues in biology and in medicine [4-8]. It is likely that microarrays will continue to be used to explore various biological phenomena. The basic underlying principle involves spotting DNA fragments either derived from polymerase chain reaction or preparation of plasmids or oligonucleotides at high density (~10,000–25,000 spots on a glass slide of 25 mm × 75 mm) representing the probes of the genes under study. The surface on which the DNA fragments or oligonucleotides are spotted is usually glass slides coated with poly-L-lysine or amino alkyl silane that serve to improve adherence of DNA to the surface. Uniform spotting at high density requires robotic operation and a variety of robots are now available for spotting [9]. The robots employed for the preparation of microarrays are of the cartesian type with movement in x-y-z direction.

A critical aspect of microarray production is the design considering space optimization to produce high-density arrays for a given set of samples and replicates. The softwares generally supplied with robotic spotters translate user input parameters into a set of instructions in robot language for printing arrays. These softwares do not offer design capabilities in which spotting parameters and grid configurations can be chosen for a given set of samples and replicates. Presently various solutions have to be derived manually in most academic laboratories. We have developed a user-friendly software 'ArrayD' that can be used by experts and novice alike to fill this gap to simplify and aid in rapid design. ArrayD offers a variety of design solutions given a set of requirements: Number of gene probes, number of replicates, and the source plate (384 well or 96 well). Because the algorithm implemented in ArrayD is inherently simple and uses fundamental principles of robot operation, the design solutions offered by ArrayD are universally applicable to any system. The choice of a design solution would be governed by the spotting chemistry and the humidity used in addition to elegant appearance. The hallmark of ArrayD is its overall simplicity and the variety of alternative designs it offers for users to decide on choosing the appropriate spotting parameters. The multiple design solutions offered by ArrayD provides a wide range of arrays from compact to loosely spaced spots as well as convenient grid patterning, which can be user selected for printing.

### Implementation

ArrayD program is developed in C and can be compiled and operated on UNIX V5.1, IRIX 5.1 and Red Hat Linux 7.0 (or higher) operating systems. A companion computer program ArraySolution was developed in Perl (Practical Extraction Report Language) version 5.6.1 and can be implemented on any UNIX or Linux operating system.

#### Inputs to be defined for ArrayD

(a) Type of source plate to be used

The input parameter toggles between either 96 well or 384 well plate.

(b) Number of gene probes to be printed

The number of gene probes including positive and negative controls and blanks.

(c) Number of replicates

The number of replicates of each gene probe. Although the most common pattern chosen is duplicates, users can choose any number of replicates. Replicates are assumed to be printed in the Y axis.

(d) Number of pins to be used for printing

This parameter relates to time taken for printing the slides and the number of spots arrayed per slide. The number of pins in X-axis and Y-axis need to be specified. The type of pins used is assumed to be stealth pins, which are widely used. It is not necessary to specify pin type for ArrayD. Instead, this aspect is considered in the printing software according to the pin type used for implementing a particular design.

## Results and discussion

**Figure 1:**
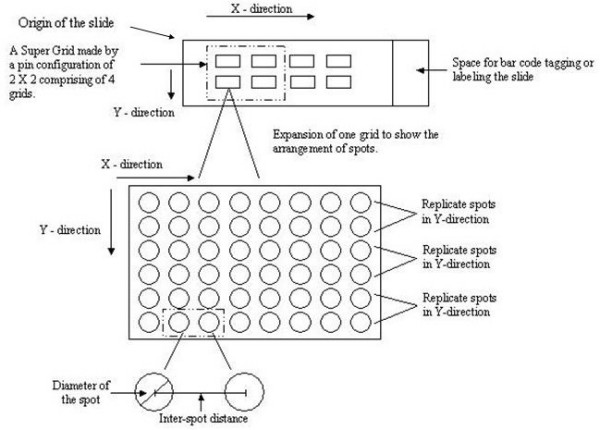
A general layout of microarray. In this example, a 2 × 2 pin configuration for 192 samples and 384 spots (duplicate) were considered. The origin is marked. Each pin prints one grid. One Super grid comprises of 4 grids, two in each axis. The number of super grids in X-direction is 2 and the number of super grids in Y-direction is 1. The spot pattern of a portion of one grid is zoomed for clarity. Replicates are spotted adjacent to each other in Y-direction. The number of samples in each grid is 24 and the total number of spots is 48. Two spots are further zoomed to show the diameter of the spot and the inter-spot distance.

A general microarray design layout is displayed in Figure 1. ArrayD accepts standard slide dimensions (25 mm × 75 mm), conceptualizes the spotting area to be 50 mm × 22 mm to provide space for barcode labeling and for appropriate placement of coverslip over the print area.

**Figure 2:**
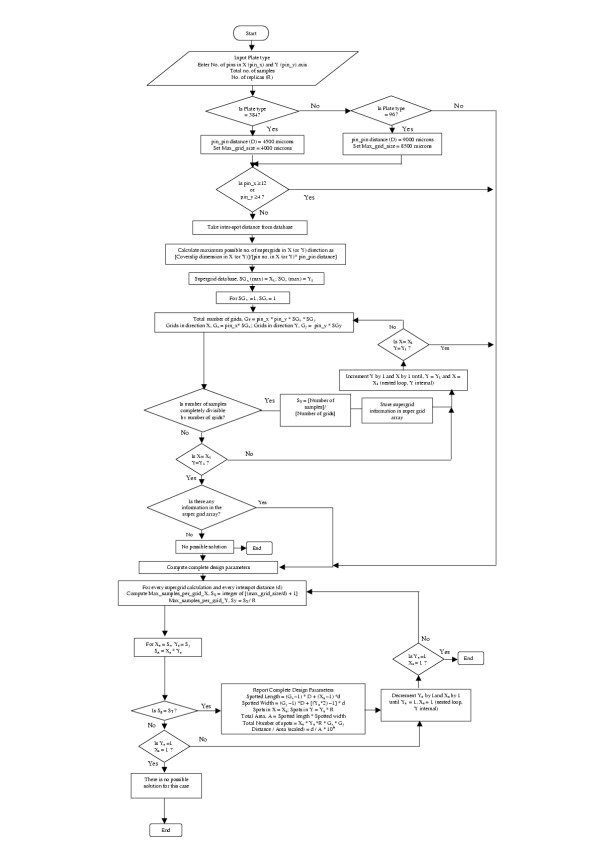
The flowchart of algorithm implemented in ArrayD to compute all possible design solutions for a given input parameters. The program first validates the given input parameters and then calculates the grid configuration. Note that in the case of 384 well plate type the pin-to-pin distance is 4500 microns on the print-head and therefore the max-grid-size is set at 4000 microns, which is 500 microns less than the upper limit (4500 microns). Similarly for 96 well plate type, the max-grid-size is set at 8500 microns (500 microns less than the upper limit 9000 microns). Maximum number of pins in the print head is taken as 48 and conforms to most printing robots. The spot distance database has inter-spot distances of 300, 250, 220, 200, 190, 180 and 170 microns. Users can expand this database. The variables used in the flowchart: pin_x: pin number in X-direction; pin_y: pin number in Y-direction; R: number of replicates; D: pin-to-pin distance; d: inter-spot distance; SG_x _(max): maximum possible number of super grids in X-direction; SG_y _(max): maximum possible number of super grids in Y-direction; G_T_: Total number of grids in the slide; G_x_: Total Number of Grids in X-direction; G_y_: Total Number of grids in Y-direction; S_T_: Maximum possible number of samples in each grid; S_x_: Maximum possible number of samples in each grid in X-direction; S_y_: Maximum possible number of samples in each grid in Y-direction; X_s_: number of samples in each grid in X-direction for a solution; Y_s_: number of samples in each grid in Y-direction for a solution; S_g_: Total number of samples per grid for a solution; A: Total area covered by the microarray on the slide.

The reference direction of the robot for picking probes from source plate is left → right followed by top → down; the printing direction is top → down followed by left → right. Replicates are considered to be spotted in Y-axis (Figure 1). After the user has entered the parameters, the software generates a text file called 'solution.txt' that carries possible alternative array design parameters for the given input. The algorithm implemented in ArrayD is displayed in Figure 2.

The program first validates the input given by the user for appropriate number of pins in each direction and the plate type to be used. For a valid input, ArrayD calculates maximum possible number of super grids in X (or Y) direction based on the coverslip dimensions, pin number in X (or Y) direction and pin-to-pin distance (Figure 2). The coverslip dimensions have been set in the program as 50 mm × 22 mm for the longest size coverslip that can be effectively used during hybridization. The pin-to-pin distance is fixed at 4500 microns in the print head for 384 well plates and at 9000 microns for 96 well plates.

ArrayD uses a predefined inter-spot distance database. Design solutions of ArrayD encompass various inter-spot distances that would be compatible with different spotting chemistries and conditions of humidity. We have used inter-spot distances of 170 μm, 180 μm, 190 μm, 200 μm, 220 μm, 250 μm and 300 μm. This database can be expanded to incorporate even lower inter-spot distances for use with other spotting chemistries by simple modification. We chose 170 μm as least distance based on several trial experiments in our laboratory using 50% DMSO as spotting solution and SMP3 pin type. In our experience, a minimum inter-spot distance of 200 microns works best with 50% DMSO at 40% – 50% humidity at 25°C.

The options for the inter-spot distance currently offered by the software can work successfully for SMP2, SMP2B, SMP2XB, SMP2.5, SMP2.5B, SMP2.5XB, SMP3, SMP3B, SMP3XB, SMP4, SMP4B, SMP4XB, SMP5, SMP5B and SMP5XB stealth pins (See Table 1). For each possible super grid configuration, the number of grids in each direction is optimized based on the number of gene probes (samples) input by the user as shown in figure 2.

### Design solutions offered by the program

Alternative array designs for a given set of input parameters are ranked on the basis of 'Distance area ratio' that describes the area covered by the array for each design. The array design spanning least area is ranked highest. This strategy allows for applying the labeled target sparingly. Subsequently, an easy report in tabular form can be generated by feeding the output data file from ArrayD into the companion Perl program 'ArraySolution.pl', which classifies array solutions into 'Square', 'Rectangle (Horizontal bar)', or 90° rotated 'Rectangle (Vertical column)' based on the geometry of a given design solution. If the number of grids are equal in both the direction we have a 'Square' design. In all other cases we obtain a 'Rectangle' design, which can be either of two types: the long side of the array is parallel to the length (Horizontal) or the width (Vertical) of the slide. The output of ArraySolution is a tab-delimited text file called 'filename.solution' where filename corresponds to the input name of the file carrying design solutions. The tabular report consist of Number of super grids in X – direction, Number of super grids in Y – direction, Number of spots per grid in X – direction, Number of spots per grid in Y – direction, Distance between two spots (in microns), Distance Area ratio and geometry of design (Square or Rectangle). This can aid users to decide on a particular design solution based on space optimization and elegant appearance.

**Figure 3:**
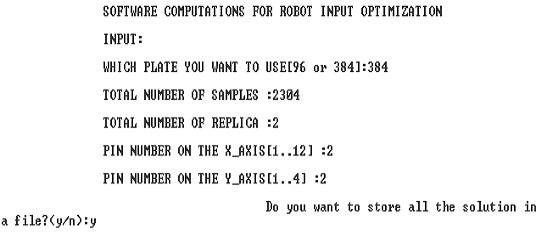
Screenshot of input parameters to ArrayD. Five inputs (Plate type (384 or 96), total number of samples, total number of replicates, pin number in X-axis and pin number in Y-axis) are fed to the program sequentially.

**Figure 4:**
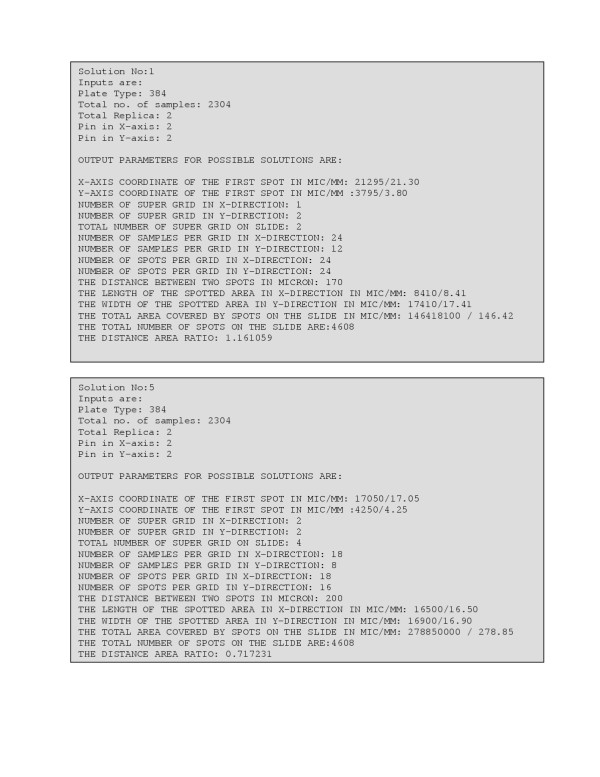
A sample run of ArrayD. The inputs given to the program were: type of plate-384; number of samples-2304; number of replicates-2; number of pins in X-axis-2; number of pins in Y-axis-2. A total of 67 solutions were offered. Two solutions are shown: top ranking solution with inter-spot distance 170 μm and an alternative design solution with inter-spot distance 200 μm. The ArrayD output was subsequently fed to the companion program 'ArraySolution' to classify each design based on its geometry and the report generated is displayed in Table 2 [see Additional file 1].

An example of a sample run is provided in Figure 3. The number of gene probes (including controls and blanks) to be spotted using a 2 by 2 pin configuration in X-Y axis is fed as 2304 (Figure 3). The gene probes have to be spotted in duplicates so the total number of spots on the slide would be 4608. The program provides 67 different array designs for various inter-spot distances, number of grids and number of spots in each grid. Two examples of different solutions are presented in Figure 4. In this example, the first solution is ranked highest with inter-spot distance of 170 microns and a 24 × 24 grid pattern with 4 grids in Y axis and 2 grids in X axis. An alternative solution provides a design with a higher inter-spot distance of 200 microns and 18 × 16 grid pattern with 4 grids each in X axis and Y axis. The first solution can be used in conditions when humidity is low and the spotting solution does not absorb moisture and spread after printing. The second solution is more appropriate for printing samples in 50% DMSO. The classification of all design solutions based on the geometries obtained from ArraySolution is displayed in Table 2 [see Additional file 1].

## Conclusion

We have developed a simple and rapid software ArrayD that offers various design solutions of designing microarrays for a specific set of user-defined requirements.

## Availability and requirements

The source code and the executable file for ArrayD and ArraySolution programs are freely available and can be downloaded from our website [11]. The source code can be compiled and executed on Unix v 5.1, or IRIX v 5.1 or Red Hat Linux v 7.0 (or higher). The executable files can be downloaded for Windows platform (Windows 98/NT/XP/2000). Further information can be requested by sending e-mail to ramu@igib.res.in or ramucbt@yahoo.com.

## Authors' contributions

AS explained the operational details to software expert, did the experimentation, maintenance and testing of the software. GPS did the basic software writing and implementation of 'ArrayD'. VKS prepared the code for the companion program 'ArraySolution' to classify the solutions on the basis of design geometry. SR is the Group Leader generating demand, sourcing and linking people, explaining the concepts, testing, critical examination, presentation of data, providing salary through grants.

## Supplementary Material

Additional File 1 Report generated by ArraySolution. A total of 67 different design solutions were classified into three categories of Square, Rectangle (Horizontal) and Rectangle (Vertical) as mentioned in text. The detailed design parameters including number of supergrids in X and Y direction, number of spots per grid in X and Y direction, distance between the spots, distance area ratio and geometry of design are provided for preparing elegant microarrays. Click here for file

## Figures and Tables

**Table 1:** Stealth pins for implementing various design solutions offered by ArrayD

Stealth Pin Catalog Number ^a^	Spot Diameter ^b ^(μm)	Minimum spot spacing ^c ^(μm)
SMP2	62.5	90
SMP2B	70	100
SMP2XB	80	110
SMP2.5	85	100
SMP2.5B	90	110
SMP2.5XB	110	130
SMP3	100	120
SMP3B	110	135
SMP3XB	125	150
SMP4	135	160
SMP4B	145	175
SMP4XB	160	190
SMP5	165	200
SMP5B	185	215
SMP5XB	200	240

^a^: The catalog numbers of pins according to Telechem Inc., USA.

^b^: The spot diameter (in microns) indicates the size of the spot after printing microarrays.

^c^: The minimum inter-spot distance that can be achieved for each of the respective pins to avoid overlapping or smudging of the spots. Data sourced from Arrayit web site [10].
