# Supplementary material for: ArrayD: A general purpose software for Microarray design
Source: BMC Bioinformatics. 2004 Oct 2;5:142. doi: 10.1186/1471-2105-5-142 (PMC524372; doi:10.1186/1471-2105-5-142)
Supplement: Additional File 1 — Report generated by ArraySolution. A total of 67 different design solutions were classified into three categories of Square, Rectangle (Horizontal) and Rectangle (Vertical) as mentioned in text. The detailed design parameters including number of supergrids in X and Y direction, number of spots per grid in X and Y direction, distance between the spots, distance area ratio and geometry of design are provided for preparing elegant microarrays. [file 1471-2105-5-142-S1.pdf]

Table 2: Report generated by ArraySolution[a].

| Solution No | Number of super grid in x-direction | Number of super grid in y-direction | Number of spots per grid in x-direction | Number of spots per grid in y-direction | Distance between two spots in microns[b] | Distance area ratio | Geometry of design[c] |
|---|---|---|---|---|---|---|---|
| 1 | 1 | 2 | 24 | 24 | 170 | 1.16 | Rectangle(Vertical column) |
| 2 | 2 | 1 | 24 | 24 | 170 | 1.16 | Rectangle(Horizontal bar) |
| 3 | 2 | 2 | 18 | 16 | 220 | 0.76 | Square |
| 4 | 2 | 2 | 16 | 18 | 220 | 0.76 | Square |
| 5 | 2 | 2 | 18 | 16 | 200 | 0.72 | Square |
| 6 | 2 | 2 | 16 | 18 | 200 | 0.72 | Square |
| 7 | 2 | 2 | 18 | 16 | 190 | 0.69 | Square |
| 8 | 2 | 2 | 16 | 18 | 190 | 0.69 | Square |
| 9 | 2 | 2 | 18 | 16 | 180 | 0.67 | Square |
| 10 | 2 | 2 | 16 | 18 | 180 | 0.67 | Square |
| 11 | 2 | 2 | 24 | 12 | 170 | 0.64 | Square |
| 12 | 2 | 2 | 18 | 16 | 170 | 0.64 | Square |
| 13 | 2 | 2 | 16 | 18 | 170 | 0.64 | Square |
| 14 | 2 | 2 | 12 | 24 | 170 | 0.64 | Square |
| 15 | 3 | 1 | 24 | 16 | 170 | 0.81 | Rectangle(Horizontal bar) |
| 16 | 3 | 1 | 16 | 24 | 170 | 0.81 | Rectangle(Horizontal bar) |
| 17 | 3 | 2 | 16 | 12 | 250 | 0.57 | Rectangle(Horizontal bar) |
| 18 | 3 | 2 | 12 | 16 | 250 | 0.57 | Rectangle(Horizontal bar) |
| 19 | 3 | 2 | 16 | 12 | 220 | 0.53 | Rectangle(Horizontal bar) |
| 20 | 3 | 2 | 12 | 16 | 220 | 0.53 | Rectangle(Horizontal bar) |
| 21 | 3 | 2 | 16 | 12 | 200 | 0.49 | Rectangle(Horizontal bar) |
| 22 | 3 | 2 | 12 | 16 | 200 | 0.49 | Rectangle(Horizontal bar) |
| 23 | 3 | 2 | 16 | 12 | 190 | 0.47 | Rectangle(Horizontal bar) |
| 24 | 3 | 2 | 12 | 16 | 190 | 0.47 | Rectangle(Horizontal bar) |
| 25 | 3 | 2 | 16 | 12 | 180 | 0.45 | Rectangle(Horizontal bar) |
| 26 | 3 | 2 | 12 | 16 | 180 | 0.45 | Rectangle(Horizontal bar) |
| 27 | 3 | 2 | 24 | 8 | 170 | 0.41 | Rectangle(Horizontal |

| | | | | | | | |
|---|---|---|---|---|---|---|---|
| | | | | | | | bar) |
| 28 | 3 | 2 | 16 | 12 | 170 | 0.41 | Rectangle(Horizontal bar) |
| 29 | 3 | 2 | 12 | 16 | 170 | 0.41 | Rectangle(Horizontal bar) |
| 30 | 3 | 2 | 8 | 24 | 170 | 0.41 | Rectangle(Horizontal bar) |
| 31 | 4 | 1 | 18 | 16 | 220 | 0.77 | Rectangle(Horizontal bar) |
| 32 | 4 | 1 | 16 | 18 | 220 | 0.77 | Rectangle(Horizontal bar) |
| 33 | 4 | 1 | 18 | 16 | 200 | 0.73 | Rectangle(Horizontal bar) |
| 34 | 4 | 1 | 16 | 18 | 200 | 0.73 | Rectangle(Horizontal bar) |
| 35 | 4 | 1 | 18 | 16 | 190 | 0.72 | Rectangle(Horizontal bar) |
| 36 | 4 | 1 | 16 | 18 | 190 | 0.72 | Rectangle(Horizontal bar) |
| 37 | 4 | 1 | 18 | 16 | 180 | 0.70 | Rectangle(Horizontal bar) |
| 38 | 4 | 1 | 16 | 18 | 180 | 0.70 | Rectangle(Horizontal bar) |
| 39 | 4 | 1 | 24 | 12 | 170 | 0.61 | Rectangle(Horizontal bar) |
| 40 | 4 | 1 | 18 | 16 | 170 | 0.61 | Rectangle(Horizontal bar) |
| 41 | 4 | 1 | 16 | 18 | 170 | 0.61 | Rectangle(Horizontal bar) |
| 42 | 4 | 1 | 12 | 24 | 170 | 0.61 | Rectangle(Horizontal bar) |
| 43 | 4 | 2 | 12 | 12 | 300 | 0.51 | Rectangle(Horizontal bar) |
| 44 | 4 | 2 | 12 | 12 | 250 | 0.43 | Rectangle(Horizontal bar) |
| 45 | 4 | 2 | 9 | 16 | 250 | 0.43 | Rectangle(Horizontal bar) |
| 46 | 4 | 2 | 18 | 8 | 220 | 0.39 | Rectangle(Horizontal bar) |
| 47 | 4 | 2 | 12 | 12 | 220 | 0.39 | Rectangle(Horizontal bar) |
| 48 | 4 | 2 | 9 | 16 | 220 | 0.39 | Rectangle(Horizontal bar) |
| 49 | 4 | 2 | 8 | 18 | 220 | 0.39 | Rectangle(Horizontal bar) |
| 50 | 4 | 2 | 18 | 8 | 200 | 0.36 | Rectangle(Horizontal bar) |
| 51 | 4 | 2 | 12 | 12 | 200 | 0.36 | Rectangle(Horizontal bar) |
| 52 | 4 | 2 | 9 | 16 | 200 | 0.36 | Rectangle(Horizontal bar) |

| | | | | | | | |
|---|---|---|---|---|---|---|---|
| 53 | 4 | 2 | 8 | 18 | 200 | 0.36 | Rectangle(Horizontal bar) |
| 54 | 4 | 2 | 18 | 8 | 190 | 0.35 | Rectangle(Horizontal bar) |
| 55 | 4 | 2 | 12 | 12 | 190 | 0.35 | Rectangle(Horizontal bar) |
| 56 | 4 | 2 | 9 | 16 | 190 | 0.35 | Rectangle(Horizontal bar) |
| 57 | 4 | 2 | 8 | 18 | 190 | 0.35 | Rectangle(Horizontal bar) |
| 58 | 4 | 2 | 18 | 8 | 180 | 0.33 | Rectangle(Horizontal bar) |
| 59 | 4 | 2 | 12 | 12 | 180 | 0.33 | Rectangle(Horizontal bar) |
| 60 | 4 | 2 | 9 | 16 | 180 | 0.33 | Rectangle(Horizontal bar) |
| 61 | 4 | 2 | 8 | 18 | 180 | 0.33 | Rectangle(Horizontal bar) |
| 62 | 4 | 2 | 24 | 6 | 170 | 0.30 | Rectangle(Horizontal bar) |
| 63 | 4 | 2 | 18 | 8 | 170 | 0.30 | Rectangle(Horizontal bar) |
| 64 | 4 | 2 | 12 | 12 | 170 | 0.30 | Rectangle(Horizontal bar) |
| 65 | 4 | 2 | 9 | 16 | 170 | 0.30 | Rectangle(Horizontal bar) |
| 66 | 4 | 2 | 8 | 18 | 170 | 0.30 | Rectangle(Horizontal bar) |
| 67 | 4 | 2 | 6 | 24 | 170 | 0.30 | Rectangle(Horizontal bar) |

[a]: The output of ArrayD for a sample run (Figure 4) were fed to ArraySolution. A total of 67 different design solutions were classified into three categories of Square, Rectangle (Horizontal) and Rectangle (Vertical) as mentioned in text.
[b]: The inter-spot distances (in microns) according to the database in ArrayD.
[c]: Of the total 67 design solutions, 12 were classified as ‘Square’ and 55 as ‘Rectangle’ (54 Horizontal, 1 Vertical).
